# Potential Utilisation of *Theobroma cacao* Pod Husk Extract: Protective Capability Evaluation Against Pollution Models and Formulation into Niosomes

**DOI:** 10.21315/tlsr2024.35.2.6

**Published:** 2024-07-31

**Authors:** Erika Chriscensia, Joshua Nathanael, Urip Perwitasari, Agus Budiawan Naro Putra, Shakila Angjaya Adiyanto, Pietradewi Hartrianti

**Affiliations:** 1 Department of Pharmacy, School of Life Sciences, Indonesia International Institute for Life Sciences (i3L), Jl. Pulomas Barat No. Kav. 88, RT.4/RW.9, Kayu Putih, Kec. Pulo Gadung, 13210 Jakarta, Indonesia; 2 Research Centre for Applied Microbiology, National Research and Innovation Agency (BRIN), 16911 Cibinong, Indonesia; 3 Research Centre for Pharmaceutical Ingredients and Traditional Medicine, National Research and Innovation Agency (BRIN), 16911 Cibinong, Indonesia

**Keywords:** Cytoprotective, Niosome, Antioxidant, *Theobroma cacao* Pod Husk, Pollution, Cell Culture

## Abstract

*Theobroma cacao* L. beans have long been used for food and medicinal purposes. However, up to 52%–76% of *Theobroma cacao* L. fruit comprises its husk, which are regarded as waste and oftentimes thrown away. In fact, cocoa pod husks actually possess a high antioxidant capacity. Antioxidants can be used to fight free radicals that are produced by environmental pollution. In order to simulate the effects of pollution, H^2^O^2^ and cigarette smoke extract models were used respectively. However, the antioxidant properties are limited on the skin due to poor penetration. Hence, in order to increase the topical penetration, cocoa pod husk extract (CPHE) was also formulated into niosomes thereafter. CPHE was characterised using total phenolic content, total flavonoid content and three antioxidant assays. After that, cytotoxicity and cytoprotective assay were conducted on HaCaT cells, which represent the skin epidermis. CPHE was then formulated into niosomes subjected to stability and penetration studies for three months. CPHE was shown to contain 164.26 ± 1.067 mg GAE/g extract in total phenolic content and 10.72 ± 0.32 mg QCE/g extract in total flavonoid content. In addition, our results showed that CPHE possesses similar antioxidant capacity through 2,2-diphenyl-1-picrylhydrazyl (DPPH) assay, around eight-fold less through ABTS assay and approximately twelve-fold less through Ferric reducing power (FRAP) assay. The extract also showed comparable cytoprotective properties to that of standard (ascorbic acid). The niosome formulation was also able to increase the penetration compared to unencapsulated extract, as well as possess a good stability profile. This showed that CPHE, in fact, could be repurposed for other uses other than being thrown away as waste.

**Highlights**

- *Theobroma cacao* pod husk extract possessed a total phenolic content of164.26 ± 1.067 mg GAE/g extract and a total flavonoid content of 10.72 ±0.32 mg QCE/g extract. Based on antioxidant capacity, the extract possessedsimilar antioxidant capacity with ascorbic acid through 2,2-diphenyl-1-picrylhydrazyl (DPPH) assay, approximately eight-fold less through 2, 2′-azino-bis-3-ethylbenzothiazoline-6-sulfonic acid (ABTS) assay and approximatelytwelve-fold less through Ferric reducing power (FRAP) assay.
- The extract showed less cytotoxicity than ascorbic acid and comparable results to that of ascorbic acid against hydrogen peroxide and cigarette smoke extractpollution models, at concentrations from 6.25 ppm and 12.5 ppm, respectively.
- Niosome F1 (4:1 soy lecithin to spam ratio) showed the best extractencapsulation efficiency at 97.08 ± 3.85%. All formulations showed stabilityover a 3-month period. F1 also showed the best penetration properties withmaximum flux at 4 h timepoint with the value of 653.27 ± 21.42 μg/h.

## INTRODUCTION

*Theobroma cacao* L. beans have long been used for food and medicinal purposes, and cocoa beans are processed into many types of products, such as chocolate, cocoa powder and cocoa butter, with many varieties in each category. However, a large part of cocoa food waste is from its pod husk, which comprises 52% to 76% of the whole cocoa fruit (Yusof *et al*. 2016). Therefore, there have been a lot of attempts to repurpose cocoa waste. In fact, cocoa pod husks actually contain high antioxidants, as well as many phytochemicals which are also constituents of the phytochemicals in cocoa beans (Baharum *et al*. 2014; Felice *et al*. 2020). This suggests that cocoa pod husks have a great potential to be utilised for other purposes as antioxidants.

Antioxidants are mainly utilised to protect against oxidative stress (Poljšak & Dahmane 2012). The effects of oxidative damage on the skin are based on the free radical theory of aging. This theory states that aging is caused by the accumulation of damage from free radicals on the cells and connective tissues (Harman 1956). Antioxidants have also been suggested to play an important role in protecting the cells from oxidative damage by various other mechanisms (Lobo *et al*. 2010; Poljšak & Fink 2014). Therefore, many anti-aging products contain antioxidants to fight these free radicals from pollutants and prevent aging.

One of the major sources of exogenous free radicals is air pollution. Out of the six major air pollutants classified by the WHO, particulate matter (PM) is shown to contribute hugely in causing diseases caused by air pollution (Crinnion 2017; Manisalidis *et al*. 2020). PM is composed of tiny matters and can be represented in a lot of models in research, for example as heavy metal model (Xu *et al*. 2021), urban diesel model (Lou *et al*. 2022), PM standard model (Li *et al*. 2017), exposure to ionising radiation (Qian *et al*. 2020) and cigarette smoke model (Gellner *et al*. 2016). Cigarette smoke in particular contains a mixture of tiny matters and gas ranging between 0.1 μm and 1 μm (Braun *et al*. 2019). This makes cigarette smoke one of the most dangerous pollutants because it can penetrate deeper into the skin, be absorbed into the systemic circulation and cause severe health effects (K. H. Kim *et al*. 2015). Therefore, cigarette smoke is used to represent particulate matter pollution models.

Another source of free radicals that have been commonly used to represent oxidative damage is hydrogen peroxide (H_2_O_2_). A lot of reactions that generate free radicals, mainly ROS, are connected to hydrogen peroxide (Griendling *et al*. 2016; Kurutas 2015). Due to this, the H_2_O_2_ pollution model can be used to illustrate the general representation of oxidative damage. Hence, the cigarette smoke and H_2_O_2_ model were constructed to simulate the effects of environmental pollution. The experiments on these pollution models are done on HaCaT cells, immortalised keratinocytes which represent up to 95% of the outermost skin barrier, the epidermis (Colombo *et al*. 2017).

Oxidative stress occurs inside the human skin especially on the inner layer of epidermis to the dermis layer (Rinnerthaler *et al*. 2015). On the other hand, extracts cannot easily penetrate the skin due to hydrophilic nature. The stratum corneum of epidermis is a resilient barrier towards hydrophilic substances. Therefore, formulation of a drug carrier is highly favourable to enhance topical penetration. Nanoparticles are one of the methods to improve topical penetration, which include liposome, niosome, nanocrystal, nanocapsule, nanosphere, nanoemulsion, solid lipid nanoparticle and nanostructured lipid carrier (Ghasemiyeh & Mohammadi-Samani 2020). These nanoplatforms have their own advantages and disadvantages. As this study focuses on extract encapsulation, vesicular nanocarriers such as liposome and niosome have been proven to possess higher encapsulation efficiency than other nanoplatforms (Ghasemiyeh & Mohammadi-Samani 2020). Liposome is known to be a suitable formulation to enhance penetration on drugs (Bakonyi *et al*. 2018). However, liposome shows stability issues due to its phospholipid characteristic, which makes it easily degrade with a low chemical stability (Ge *et al*. 2019). Hence, its counterpart, niosome, has emerged as a better alternative. Formulated using non-ionic surfactant and cholesterol, niosome is able to enhance formulation stability due to the non-toxic, stable non-ionic surfactant and even smaller particle size, thus having enhanced penetration (Bartelds *et al*. 2018). The hydrophilic-lipophilic balance (HLB) value of non-ionic surfactant highly contributes to the particle size of niosome (Nowroozi *et al*. 2018). The lower HLB value of Span type surfactant enables the formulation to have smaller particle size. On the other hand, an increasing amount of cholesterol can stabilise the niosome formulation with HLB lower than 6 (Moghassemi & Hadjizadeh 2014). Therefore, in this study, niosome was used to encapsulate the *Theobroma cacao* L. pod husk extract with Span 80 and soy lecithin as the nonionic surfactant and cholesterol, respectively.

The objectives of this study were to extract *Theobroma cacao* L. pod husk, measure its antioxidant activity and observe its cytoprotective effects on immortalised human keratinocytes HaCaT cells upon exposure to pollution. Thereafter, the cocoa pod husk extract was formulated into penetration-enhancing niosomes and subjected to penetration and stability studies.

## MATERIALS AND METHODS

### Materials

Gallic acid, quercetin, aluminium chloride (AlCl_3_), 2,4,6-tripyridyl-S-triazine (TPTZ), 2, 2′-azino-bis-3-ethylbenzothiazoline-6-sulfonic acid (ABTS), phosphate buffer saline (PBS) tablets and sodium bicarbonate (NaHCO_3_) were purchased from Sigma Aldrich (USA). Folin-Ciocalteu reagent, sodium hydroxide (NaOH), absolute ethanol, absolute methanol, ascorbic acid (AA), hydrogen chloride (HCl), sodium acetate (CH_3_COONa), glacial acetic acid (CH_3_COOH), ferric chloride (FeCl_3_) and hydrogen peroxide (H_2_O_2_) were purchased from Merck (Germany). Dulbeccoo’s Modified Eagle’s Medium (DMEM) powder, fetal bovine serum (FBS) and penicillin-streptomycin were purchased from Gibco (USA). Potassium acetate was purchased from Bio Basic (Canada). DPPH powder was purchased from Himedia (India). Ammonium persulfate was purchased from Loba Chemie (India). 3-(4,5-dimethylthiazol-2-yl)-5-(3-carboxymethoxyphenyl)-2-(4-sulfophenyl)-2H-tetrazolium (MTS) reagent was obtained from Promega (USA). Cigarettes used for this study was Dji Sam Soe Kretek brand which is manufactured by PT Gudang Garam Tbk, Indonesia.

### Extraction of Plant Material

The *Theobroma cacao* L. pod husk extract was obtained from Lembaga Ilmu Pengetahuan Indonesia (LIPI), South Jakarta, Indonesia. Cacao pods used in this study were from the Forastero variety of TSH-858 clone. They were collected from Kutadalom, Pesawaran Regency, Lampung Province, Indonesia (5°25’14.02”S, 105°1’0.66”E). All cacao clone, including TSH-858, planted in Pesawaran Regency, Lampung Province, had been identified by the Directorate of Estate Crops, Ministry of Agriculture, Republic of Indonesia. The morphological difference of the cacao pods from different clones collected in Lampung Province, Indonesia has also been reported elsewhere (Fahrurrozi *et al*. 2021). The extraction of *Theobroma cacao* L. pod husk was done in accordance with (Romulo *et al*. 2018) with some modifications. Briefly, 20 g of powder underwent two cycles of maceration with ethanol 70% (1:5) using an incubator shaker at 170 rpm at 25°C. After that, the solution was centrifuged at a temperature of 4°C and a speed of 5,000 G for 10 min. Subsequently, the sample was re-extracted using the same method, and then collected the filtrate. Afterward, the solvent was evaporated by using a rotary evaporator then the sample was dried with a freeze dryer (Alpha 1-2 LD Plus Christ). For the penetration study, the dried samples were diluted in 10% dimethyl sulfoxide (DMSO) in phosphate-buffered saline (PBS) solution (10% DMSO-PBS). The extracts were dissolved at four different concentrations of 50% (1:1 g/mL); 25%; 12.5%; and 6.25%. In this study, chloramphenicol was used as a positive control and 10% DMSO as a negative control.

### Evaluation of *Theobroma cacao* L. Pod Husk Extract

#### Total phenolic content

The total phenolic content of *Theobroma cacao* L. pod husk extract was measured using the Folin-Ciocalteu method according to (Ghafar *et al*. 2017). The extract was dissolved in methanol to make a concentration of 400 ppm. Two milliliters (2 mL) of the extract solution was mixed with 5 mL 7.5% Folin-Ciocalteu reagent inside a test tube and was incubated in a dark condition for 8 min. After that, 4 mL of 1 % NaOH was added to the solution. The absorbance of the samples was measured using a UV-Vis spectrophotometer (Shimadzu 1280) at 730 nm. A standard curve was constructed with gallic acid and the results were expressed as milligrams of gallic acid equivalent GAE/g extract.

#### Total flavonoid content

The total flavonoid content of *Theobroma cacao* L. pod husk extract was measured using the aluminium chloride method according to (Ghafar *et al*. 2017). The extract was dissolved in ethanol to make a concentration of 1,000 ppm. Two milliliters (2 mL) of the extract was transferred into a test tube. A total of 0.1 mL 10% AlCl_3_, 0.1 mL 1 M CH_3_COOH and 2.8 mL of distilled water were incubated for 30 min in the dark. The absorbance of the samples was measured using a UV-Vis spectrophotometer (Shimadzu 1280) at 415 nm. A standard curve was constructed using quercetin and the results were expressed as milligrams of quercetin equivalent QE/g extract.

#### Antioxidant capacity assays

##### (a)2,2-diphenyl-1-picrylhydrazyl (DPPH) assay

DPPH assay was done according to previously mentioned methods (Jiménez-Estrada *et al*. 2013). The antioxidants in the plant extract should be able to reduce DPPH into 1,1-diphenyl-2-picrylhydrazyl, which can absorb light at 517 nm. 0.1 mM DPPH is prepared in methanol. DPPH solution and a series of concentrations of extract and AA as the standard were made. AA was chosen as standard as it is a gold standard of antioxidant, where it is commonly recognised as a potent antioxidant (D.-O. Kim *et al*. 2002). The samples and DPPH solution were mixed at 1:1 ratio, then incubated in the dark and at room temperature for 30 min. The absorbance was then read at 517 nm on a UV-Vis Spectrophotometer Shimadzu 1280 (Shimadzu, Japan). The free radical scavenging (%) was calculated using the following formula and the IC_50_ of the extract was calculated using non-linear regression analysis.


$$\text{Free radical scavenging ability }(\%)=\frac{Ac-As}{Ab}\times 100\%$$

where *Ac* = absorbance of control and *As* = absorbance of sample or standard.

##### (b)Ferric reducing power (FRAP) assay

FRAP assay was done according to previously mentioned methods with some modifications (Jiménez-Estrada *et al*. 2013). TPTZ reagent was made fresh (10 mM TPTZ in 40 mM HCl, 300 mM acetate buffer with pH 3.6, 20 mM FeCl_3_ solution at ratio of 1:10:1). A standard curve using AA was constructed. 100 μL of plant extract sample (1,000 ppm) was mixed with 3 mL of TPTZ reagent. The solution was incubated for 10 min at room temperature in the dark. The absorbance was measured at 593 nm on a UV-Vis Spectrophotometer Shimadzu 1280 (Shimadzu, Japan) to measure the colored product (ferrous tripyridyltriazine complex). The results were expressed as milligrams of ascorbic acid equivalents (mg AA/g).

##### (c)2,2′-azino-bis-3-ethylbenzothiazoline-6-sulfonic acid (ABTS) assay

ABTS assay was done according to previously mentioned methods with some modifications (Jan *et al*. 2013; Zheleva-Dimitrova *et al*. 2010). The radical scavenging activity in ABTS assay was determined by calculating the disappearance of the ABTS radical cation after 6 min. ABTS reagent was made by mixing ABTS and ammonium persulfate to a final concentration of 7 mM and 2.45 mM, respectively. The solution was incubated for 16 h in darkness at room temperature. The next day, ABTS radical solution was diluted to an absorbance of 0.700 at 734 nm with water on a UV-Vis Spectrophotometer Shimadzu 1280 (Shimadzu, Japan). A standard curve was made with AA as the standard. The sample and the diluted ABTS radical solution were mixed at a ratio of 1:20 v/v. The decrease in absorbance was taken after 6 min of letting the solution react in darkness at room temperature. The percentage inhibition was calculated using the following formula:


$$\text{ABTS radical scavenging activity }(\%)=\frac{Ac-As}{A\text{c}}\times 100\%$$

where *Ac* = absorbance of control and *As* = absorbance of sample or standard.

### Cigarette Smoke Extraction

**Figure 1:**
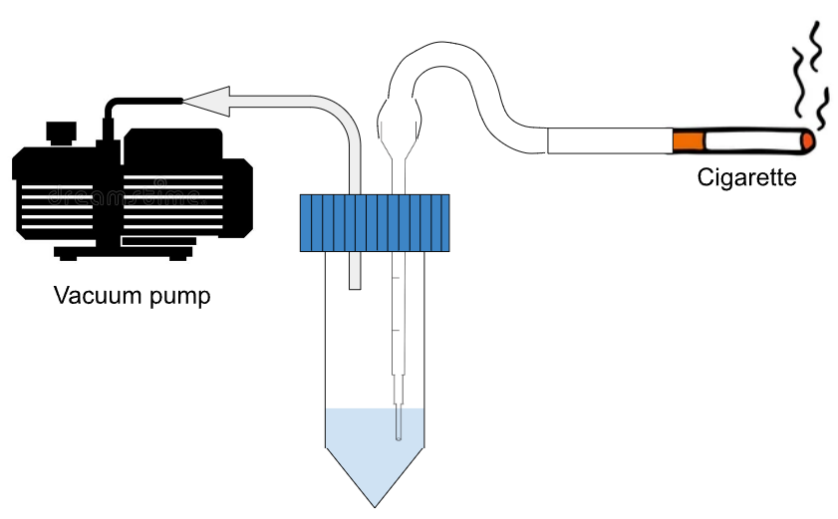
Cigarette smoke extraction apparatus illustration.

The cigarette smoke extraction was done according to previous methods (Sarkar *et al*. 2019; Yoon *et al*. 2011) as illustrated in Fig. 1. Briefly, one cigarette was extracted in 10 mL PBS by smoking the cigarette through a vacuum pump (Oil-less Piston Vacuum Pump MAJP-40V(L), RoHs, China). The cigarette smoke was passed through and bubbled into the liquid inside a 50 mL centrifuge tube. This cigarette smoke extract (CSE) solution was considered as a 100% stock solution.

### *In vitro* Assay in HaCaT Cells

#### Cytotoxicity assay

The *in vitro* cytotoxicity tests were performed according to ISO 10993-5 regarding tests for *in vitro* cytotoxicity. HaCaT cells were maintained in Dulbecco’s Modified Eagle’s Medium (DMEM) supplemented with 10% FBS and 100 μg/mL penicillin-streptomycin at 37°C in an incubator (ThermoScientific, USA) supplied with 5% CO_2_. 3-(4,5-dimethylthiazol-2-yl)-5-(3-carboxymethoxyphenyl)-2-(4-sulfophenyl)-2H-tetrazolium (MTS) assay, also known as one-step 5-(3-carboxymethoxyphenyl)-2-(4,5-dimethyl-thiazoly)-3-(4-sulfophenyl) tetrazolium (MTT) assay, was conducted to perform cytotoxicity studies on the extract against HaCaT cells *in vitro*. HaCaT cells were grown in 96-well plates with a seeding density of 1 × 10^4^ cells per well and were incubated for 24 h. At around 90% confluency, cells were treated with cocoa pod husk extract (CPHE) and AA separately. Concentrations were made ranging from 100 to 6.25 ppm of both extract and AA. The cells were treated for 24 h. Before the MTS assay, the cells were photographed by using an inverted light microscope (Inverted Microscope Primovert, Carl Zeiss) with 200× magnification. The cell viability is measured after washing the cells with DMEM twice gently. The wells that contain the treated cells were added with 100 μL cDMEM and 15 μL of MTS reagent. The cells were then incubated for 4 h in a CO_2_ incubator. Aluminium foil was wrapped around the plate to prevent light-induced degradation of MTS. The absorbance was measured at 490 nm on a plate reader (The Infinite®M200 NanoQuant, TECAN, Switzerland). Cell viability was measured in percentage according to this equation:


$$\text{Cell viability }(\%)=\frac{As-Ab}{An-Ab}\times 100\%$$

Where *As* = absorbance of sample or standard; *Ab* = absorbance of blank (DMEM only without cells) and *An* = absorbance of negative control.

The cytotoxicity of H_2_O_2_ and CSE are also observed. HaCaT cells were treated with H_2_O_2_ of concentrations ranging from 200 to 1.5625 μM and CSE extracts with concentrations ranging from 32% to 0.125%. The cell viability was then measured using MTS assay.

#### Cytoprotective assay

HaCaT cells are seeded at a density of 1 × 10^4^ cells per well in a 96-well plate for 24 h and incubated at 37°C in 5% CO_2_. H_2_O_2_ and CSE were used to induce oxidative stress in this experiment respectively. Old HaCaT cells (> 9th passage) were pre-treated with CPHE and AA for 1 h before the challenge in a 5% CO_2_ incubator at 37°C. The cells were then challenged with 6.25 μM H_2_O_2_ and 1% CSE in DMEM respectively, along with treatment of CPHE and AA respectively in each well. Cell viability measurements were conducted with MTS assay. The IC_50_ of each treatment was measured by reading the absorbance at 490 nm on a plate reader (The Infinite®M200 NanoQuant, TECAN, Switzerland) and the results were compared with negative control which were cells that were only exposed to either H_2_O_2_ or CSE. The percentage protection was calculated with this equation:


$$\text{Protection}(\%)=\frac{As-Ab}{Ap-Ab}\times 100\%$$

where *As* = absorbance of sample or standard; *Ab* = absorbance of blank (DMEM only without cells) and *Ap* = absorbance of positive control (cells that were only exposed to treatment agents).

### Formulation of Niosomes

The formulation of the niosome was done using the thin-film hydration method in order to form small nanoparticles of the formulation, with compositions as described in Table 1.

Span 80 and soy lecithin were weighed and transferred into 300 mL 96% ethanol. The solution was heated using a hot plate up to a boiling point and stirred using a homogenizer (WiseTis HG 15 A, VWR, United Kingdom). This treatment was repeated until all components were dissolved properly. The extract was added to the solution when the temperature of the solution reached approximately 60°C and dissolved using a homogeniser (WiseTis HG 15 A, VWR, United Kingdom). In addition, the sample was sonicated (Ultrasonic Bath Sonorex RK52, Buch & Holm, Denmark) for 1 h to make sure the components were fully dissolved. After sonication, the sample was subjected to a rotary evaporator (R-100 rotavapor, Buchi, Switzerland) at 60°C and 175 mbar to remove the ethanol. The evaporation was stopped until a thin film was formed at the bottom of the rotary flask. Then, 10 mL of preheated PBS (above 60°C) was added to the rotary flask and swirled. The sample was subjected to a rotary evaporator at 60°C with normal pressure (1,000 mbar) for 1 h. The niosome was collected inside a 50 mL falcon tube and incubated at room temperature for 2 h to enable swelling of the niosomes. After incubation, the sample was sonicated (Ultrasonic Bath Sonorex RK52, Buch & Holm, Denmark) for 2 h to further disperse the niosome until small unilamellar vesicles were formed. The sonicated sample was added with 0.5 % w/v of carbopol and was sonicated for a further 15 min.

### Characterisation of Niosomes

#### Encapsulation efficiency determination

The encapsulation efficiency of formulated niosome was measured using the exhaustive dialysis method. One mL (1 mL) of each niosome formulation was transferred into different dialysis bags. The sample was sealed and put in a separate beaker containing 50 mL PBS solution (pH 7.4). The solution was stirred using a magnetic stirrer for 24 h. The loose extract in the outside compartment was sampled and treated with TPC assay, then measured using UV-Vis Spectrophotometer (Shimadzu, Japan) at 730 nm. A series of standard *Theobroma cacao* L. pod husk extract was also made using TPC assay. The encapsulation efficiency (%) was calculated with this formula:


$$\text{Encapsulation efficiency }(\%)=\frac{T-F}{T}\times 100\%$$

where *T* = Total extract (ppm) and *F* = free extract in the supernatant (ppm).

#### pH measurements

The pH of each formulated niosomes was scanned using a sensitive pH meter (Starter ST3100 pH Meter, Ohaus, USA) with proper calibration. A triplicate amount of reading was done to each formulated niosomes. A pH measurement of extract dissolved in water was also done as a part of the stability analysis.

#### Surface charge measurement

The zeta potential of each formulated niosome was determined to measure the surface charge of the formulation using a zetasizer (Zetasizer Nano EZ, Malvern, United Kingdom). The result was presented as reading in millivolts.

#### Particle size measurement

The particle size of formulated niosomes was determined using the dynamic light scattering quantification method using a zetasizer (Zetasizer Nano ZS, Malvern, United Kingdom). The principle was to measure the quantity of the fluctuation of intensity that occurs during the Brownian motion of the niosomes particles. In this case, the size distribution was determined by converting the diffusion coefficient. Thus, the average niosome particle size could be measured. The data was presented in Z-average in nm.

#### Penetration study

The penetration study was done using *in vitro* Franz diffusion method. Porcine ear was used as a skin substitute (Silva *et al*. 2022). Before usage, the skin was thawed at 4°C overnight and at room temperature. 9 mL of receptor volume was used in this experiment. The prepared porcine ear was placed between the donor and receptor compartment with the epidermis facing the donor compartment. The receptor compartment was filled with PBS pH 7.4 solution with a magnetic stirrer to ensure solution uniformity. The compartment was maintained in a water bath (WNB 22, Memmert, Germany) at 37°C. The total of 1 mL of the niosome sample was administered to the donor compartment and sealed with parafilm. A triplicate of 1 mL mixture was taken at each time point and the lost volume of the receptor compartment was relieved with PBS 7.4 solution. The intervals of time when samples were taken were 30, 60, 120, 180, 240, 360 and 1440 min. The withdrawn solution was subjected to measurement using a UV-Vis spectrophotometer (Shimadzu, Japan) under TPC assay. A graph of flux was also constructed using the data to measure the rate of penetration at specific timepoints. The result was expressed as cumulative drug release (%) and calculated using these formulas:


$$\text{Amount of drug release }(\text{mg}/\text{mL})=Ct\times Dv\times \frac{DF}{1000}$$

where *Ct* = concentration at specific time point (ppm); *Dv* = dissolution bath volume (mL) and *DF* = dilution factor.


$$\text{Cumulative drug release }(\%)=\frac{Vs}{V}\times c(t-1)+c(t)$$

where *Vs* = volume of sample withdrawn (mL), *V* = bath volume (mL), *c*(*t* − 1) = concentration before specific time point (%) and *c*(*t*) = concentration at specific time point (%).

#### Stability study

The stability studies were done by measuring the encapsulation efficiency, pH measurement, zeta potential, and particle size on day 0 and day 90 of the niosome formulations.

### Statistical Analysis

The data obtained from the experiment were presented as mean ± standard deviation. All the statistical significance analyses were done using GraphPad Prism 9.3.1. The normality of data distribution was analysed by the Shapiro-Wilk test. Data that were normally distributed were analysed using one-way ANOVA, accompanied with Dunnet’s post-hoc testing, where a *p*-value of less than 0.05 was considered statistically significant. Meanwhile, data that were not normally distributed using Kruskal-Wallis test were accompanied with Dunn’s post hoc test, where a *p*-value of less than 0.05 was considered statistically significant.

For the niosome formulation, the data of encapsulation efficiency, cumulative drug release, and flux were checked for normality using the Shapiro-Wilk test. Encapsulation efficiency was analysed using one-way ANOVA with a post-hoc by Tukey’s test. The cumulative drug release and flux were analysed using two-ways ANOVA with a post-hoc test by Tukey’s test.

## RESULTS

### *Theobroma cacao* L. Pod Husk Extract Characterisation

The obtained CPHE contained a total phenolic content of 164.26 ± 1.067 mg GAE/g extract and a total flavonoid content of 10.72 ± 0.32 mg QE/g extract.

The results of three different antioxidant assays on CPHE and AA were summarised in Table 2. The generated graphs of DPPH and ABTS assays were sigmoidal curves. The inhibitory concentration (IC_50_) of both AA and CPHE was determined and calculated by non-linear regression. Meanwhile, AA showed a linear relationship with FRAP inhibition.

The IC_50_ for AA was calculated to be 13.38 ± 5.96 ppm with a maximum inhibition of 82.12 ± 0.45%, whereas CPHE was 19.20 ± 3.59 ppm, with a maximum inhibition of 79.16 ± 1.27%. This showed that between the two, AA had better antioxidant potential when tested through the DPPH assay. On the other hand, the IC_50_ of AA was estimated to be 3.46 ± 1.79 ppm, while the IC_50_ of CPHE was 24.10 ± 1.63 ppm. According to the equation obtained from AA standard curve, CPHE was estimated to contain an equivalence of 81.45 mg AA/g extract through FRAP assay.

### *In vitro* Viability of CPHE in HaCaT Cells

#### Cytotoxicity studies

**Figure 2:**
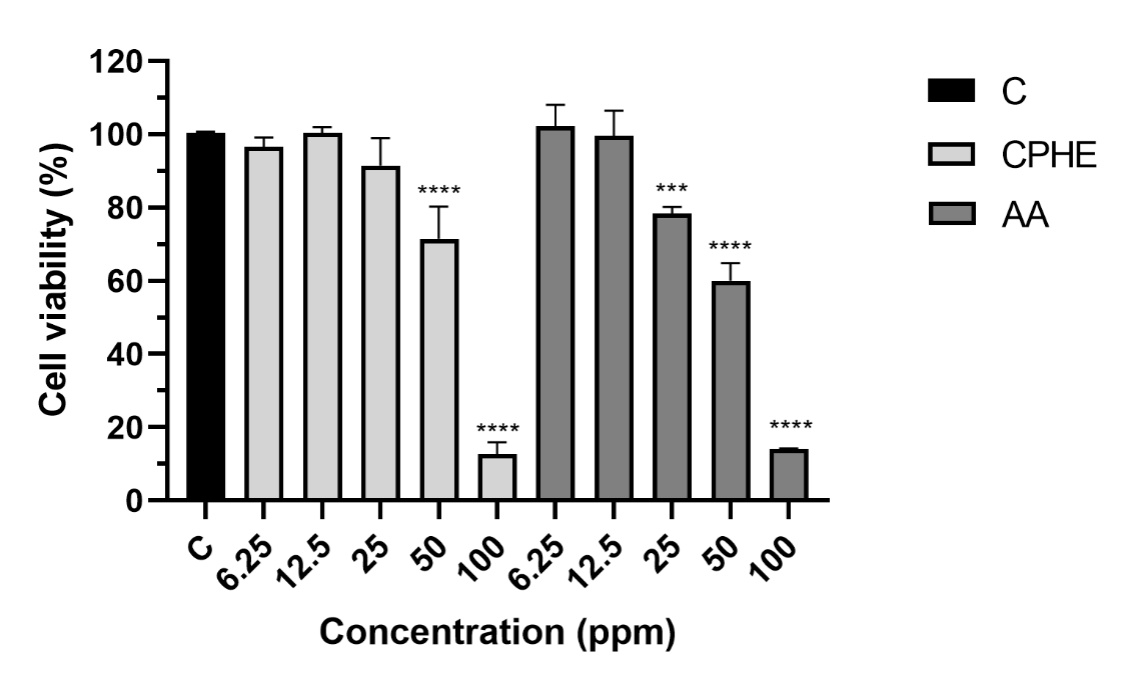
Cell viability percentage of CPHE and AA in various concentrations after 24 h. The result was compared to negative controls (C) where the cells were not treated with CPHE or AA and expressed as mean ± standard deviation of triplicates sample using one-way ANOVA, post-hoc Tukey. ***indicate statistical significant difference against negative control (*P* = 0.0005). ****indicate statistical significant difference against negative control (*P* < 0.0001).

Cytotoxicity studies were done on both treatment and insult agents. The treatment agents referred to CPHE and AA, while insult agents referred to H_2_O_2_ and CSE. The cell viability of HaCaT cells upon CPHE and AA treatments were illustrated in Fig. 2. Pictures of HaCaT cells were taken and recorded in Supplementary Table 1. According to the cell viability studies, CPHE started showing significant cytotoxic effects from 50 ppm and above, while AA started from 25 ppm and above.

#### Cytoprotective studies

**Figure 3:**
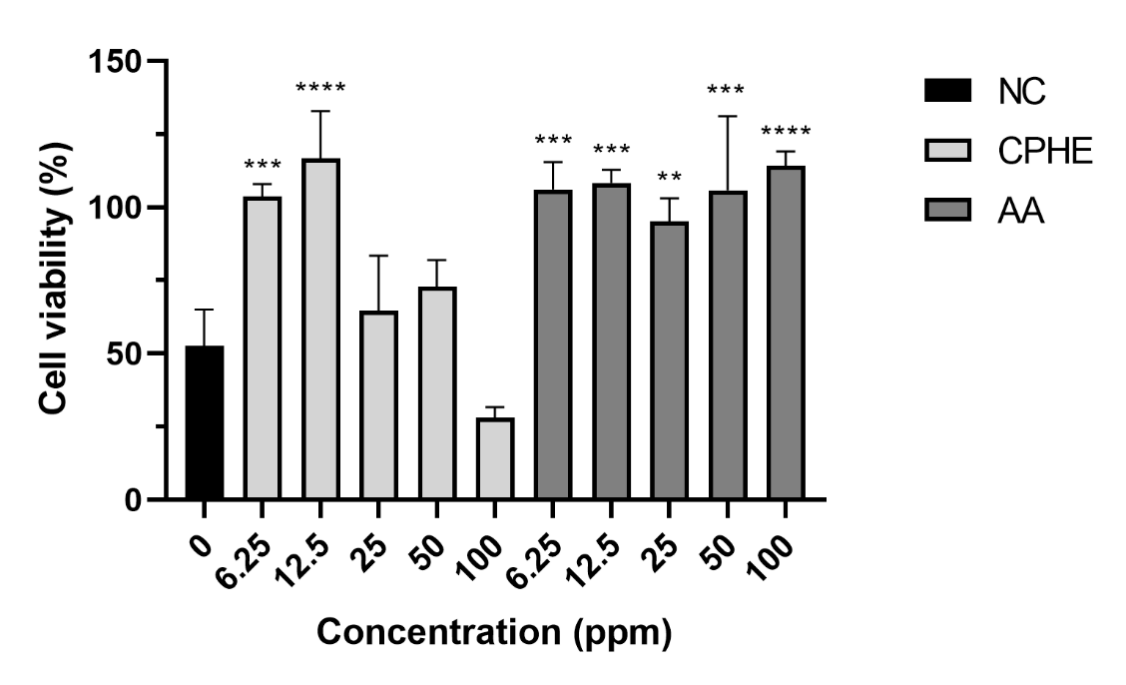
Cell viability percentage of HaCaT cells after 1 h pre-treatment of extract and 24 h extract treatment and H^2^O^2^ insult. The result was compared to negative controls (NC) where the cells were not treated with CPHE or AA and expressed as mean ± standard deviation of triplicates sample using one-way ANOVA, post-hoc Tukey. **indicate statistical significant difference (*P* < 0.01); ***indicate statistical significant difference (*P* < 0.001); ****indicate statistical significant difference (*P* < 0.0001).

**Figure 4:**
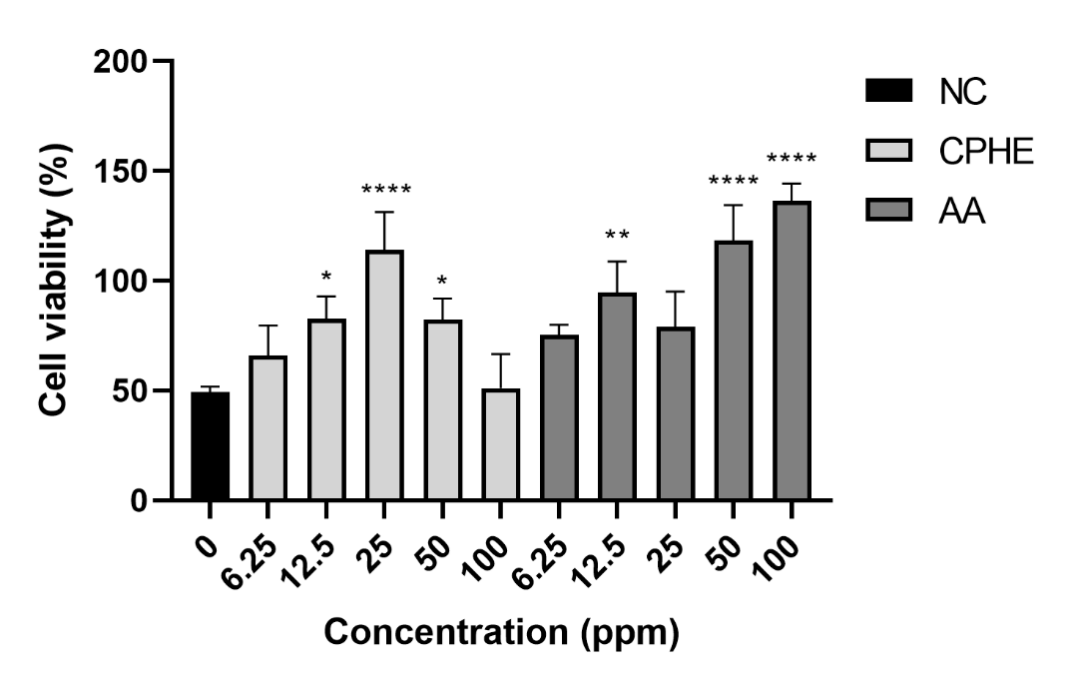
Cell viability percentage of HaCaT cells after 1 h of extract pre-treatment and 24 h extract treatment and CSE insult. The result was compared to negative controls (NC) where the cells were not treated with CPHE or AA and expressed as mean ± standard deviation of triplicates sample using one-way ANOVA, post-hoc Tukey. *indicate statistical difference (*P* < 0.1). ** indicate statistical significant difference (*P* < 0.01). **** indicate statistical significant difference (*P* < 0.0001).

The results of cytoprotective studies on H_2_O_2_ and CSE insult were depicted in Figs. 3 and 4, respectively. Pictures of HaCaT cells upon H_2_O_2_ and CSE insult were taken and recorded in Supplementary Tables 2 and 3, respectively. As a result of H_2_O_2_ insult, concentrations of 6.25 ppm and 12.5 ppm of CPHE were able to significantly protect the HaCaT cells compared to negative control, while all concentrations of AA (6.25 ppm–100 ppm) were able to significantly protect the HaCaT cells. On the other hand, concentrations of 12.5, 25 and 50 ppm of CPHE were able to significantly protect the HaCaT cells compared to negative control, while concentrations of 12.5, 50 and 100 ppm of AA were able to significantly protect the HaCaT cells.

### Niosome Formulation

The niosomes were formulated as described in Table 1. All of the final formulations were subjected to characterisation and stability test for comparison.

#### Encapsulation efficiency

**Figure 5:**
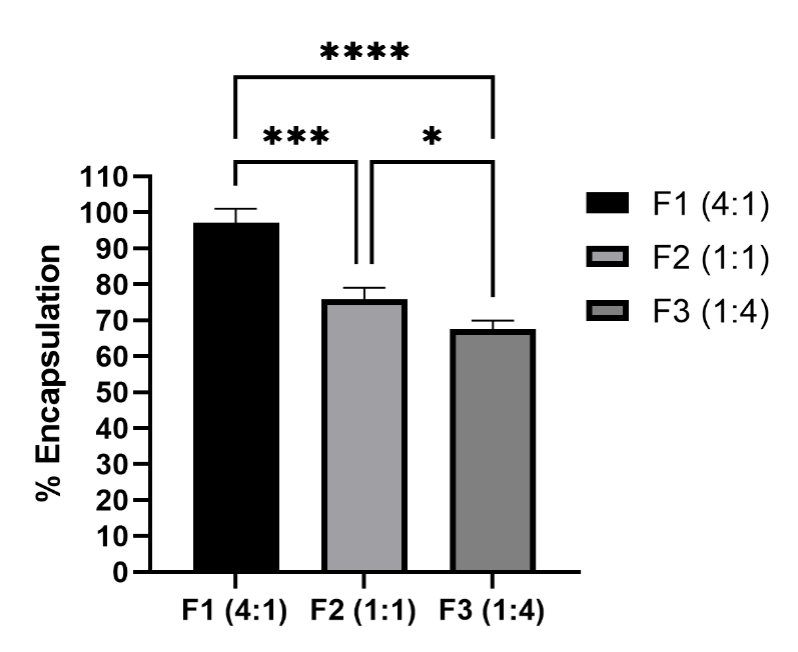
Percentage of encapsulation efficiency (*n* = 3, *p* < 0.05, One-way ANOVA, post-hoc using Tukey’s test, * indicates *p*-value < 0.05, *** indicates *p-*value < 0.001, **** indicates *p*-value < 0.0001).

The encapsulation efficiency was done to estimate the total amount of extract encapsulated in niosomes. The encapsulation efficiency (%) of different formulations were depicted in Fig. 5. F1 (4:1) has the highest encapsulation efficiency (%) with a value of 97.08 ± 3.85%. In addition, F1 (4:1) has the significant difference compared to F2 (1:1) and F3(1:4) with the value of 75.89 ± 3.17% (*p*-value < 0.001) and 67.50 ± 2.39% (*p*-value < 0.0001), respectively.

#### Stability test

The stability tests were done at day 0 and day 90 to CPHE using TPC and pH measurement. Several aspects which consist of pH, zeta potential, particle size and encapsulation efficiency were tested to test and compare each formulation stability in the different niosome formulations, as summarised in Table 3.

All of the pH resulted in small differences at day 90 when compared to the day 0 test in niosome formulations. The surface charge and particle size test of the formulation F1 (4:1) at day 0 showed the highest zeta potential and lowest particle size with the value of −21.8 ± 0.95 mV and 375.93 ± 10.75 nm, respectively. Meanwhile on day 90, all of the formulation showed lower zeta potential and higher particle size with F1 (4:1) still has the higher zeta potential and lowest particle size compared to the other formulations with the value of −19.62 ± 0.72 mV and 410.16 ± 2.43 nm, respectively. There was an increase of particle size observed in all three formulas ranging from 1.09 to 3.89%, with the least increase in F1 and highest increase in F3.

### Penetration study

**Figure 6:**
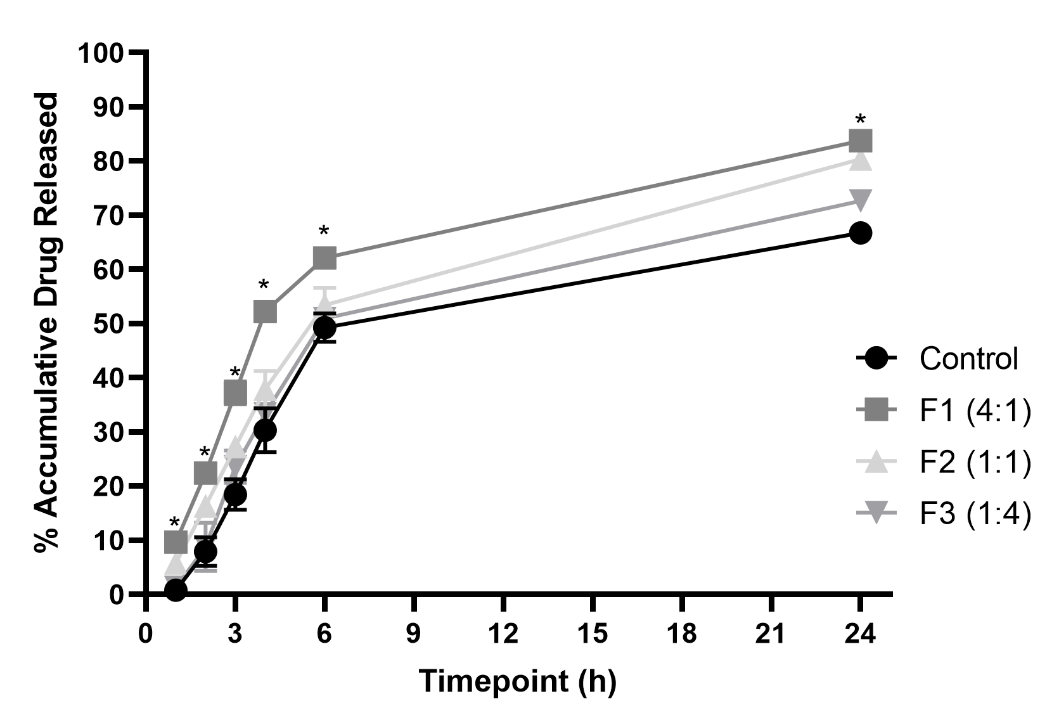
Cumulative drug release (%) over 24 h (*n* = 3, *p* < 0.05, 2-way ANOVA, post-hoc using Tukey’s test, * indicates *p*-value < 0.05 over control).

All of the formulations were tested for penetration capacity using the Franz diffusion cell apparatus on porcine ear skin as a skin model. The result showed the capability of niosome formulations to penetrate the skin barrier compared to the control of non-formulated extract. The result was reflected as cumulative drug release (%) in Fig. 6. F1 (4:1) shows the highest cumulative drug release (%) with a value of 83.75 ± 1.37%. Moreover, F1 (4:1) has the only significant difference when compared to the control solution at all time point measurements as shown in Fig. 6.

**Figure 7:**
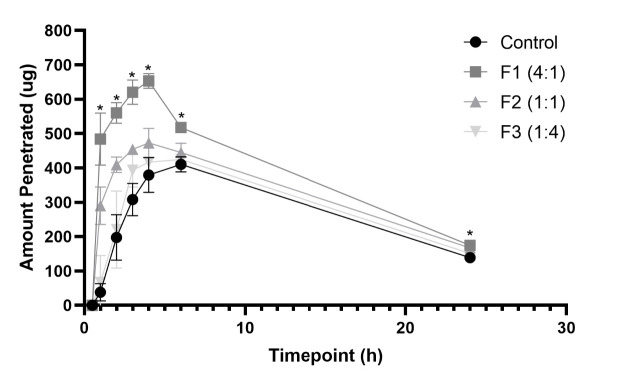
Rate of penetration or flux (mg/h) over 24 h (*n* = 3, *p* < 0.05, 2-way ANOVA, post-hoc using Tukey’s test, * indicates *p*-value < 0.05 over control).

In addition, rate of penetration (flux) was calculated and plotted into a graph against time as depicted in Fig. 7. The flux of all the niosome formulations was higher compared to the control with F1 (4:1), which showed the highest flux at 4 h timepoint with the value of 653.27 ± 21.42 ug/h. Moreover, F1 (4:1) showed a significant difference compared to control.

## DISCUSSION

### *Theobroma cacao* L. Pod Husk Extract Characterisation

#### Total phenolic content and total flavonoid content of CPHE

Phenolic content and flavonoid content were analysed in correlation with the antioxidant capacity of CPHE. Phenolic compounds themselves have phenol rings that are stabilised by resonance, rendering the compounds as a good antioxidant that has a great stability even when the compounds turn into radical compounds (Birasuren *et al*. 2013). On the other hand, a lot of studies have revealed the direct correlation between flavonoid content and antioxidant capacity (Hatami *et al*. 2014). The antioxidant mechanisms have been suggested that they can scavenge free radicals, chelate metal ions and inhibit xanthine oxidase and anti-inflammation properties (Kumar & Pandey 2013; Panche *et al*. 2016).

The phenolic and flavonoid contents of CPHE vary in different studies. These variations could be caused by different extraction methods, and the origin of the plant itself. Unlike MAE, which requires a temperature around 60 to 80°C for optimum phenolic compound extraction, room temperature may be used in maceration, decreasing the likelihood of compromising the amounts of phenolic compounds extracted (Mashuni *et al*. 2020). On the other hand, it was found that plants that grow in a high altitude, cold climate or semi-arid environment contain the highest amounts of phenolic compounds (Bernal *et al*. 2013; Kabtni *et al*. 2020). Other environmental factors also affect the phenolic content of the extract, especially from soil nutrients, foliar nutrients, as well as rainfall (Borges *et al*. 2013). Another study also suggested that variations in plant metabolite contents is attributed to the genetic variability as well (Nagahama *et al*. 2021).

#### Antioxidant potential of the extract

Three different antioxidant assays were done to measure the antioxidant activity through three different assays. Up to this date, there is no antioxidant assay that can give a definitive elucidation of a compound’s antioxidant capacity, hence why it is advisable to perform multiple assays with different mechanisms to provide a wider perspective of the antioxidant capacity (Xia *et al*. 2017).

DPPH is a nitrogen radical, different from peroxyl radicals found in lipid peroxidation. Due to the possibility of free radical termination by accepting either hydrogen radical or electron, this assay is unable to differentiate between the two (Dorsey & Jones, 2017). The maximum DPPH radical inhibition of CPHE and AA was found to be 79.16% and 82.12%, respectively. Although the concentration needed to achieve that concentration of inhibition, both extracts showed similar maximum inhibition. This showed that both CPHE and AA had strong antioxidant capacity (Lessa *et al*. 2018). An EC_50_ lower than 30 ppm by DPPH assay means that the antioxidant has a high efficiency to act as free radical scavenger, especially against superoxide anion (Abdul Karim *et al*. 2014). This means that even though CPHE possesses less potent antioxidant activity than AA, due to having it still has a high efficiency and strong antioxidant capacity to act as free radical scavenger and exhibit its antioxidant activity.

On the other hand, FRAP (Ferric reducing ability of plasma) measures the ability of compounds to reduce ferric-tripyridyltriazine (Fe(III)-TPTZ) complexes into ferrous (Fe(II)) form. The experiment showed that CPHE possesses 81.45 mg AA equivalent per gram of dried extract, which means that it took approximately 12.5 times more amount of CPHE than AA to possess similar antioxidant activity as AA.

The ABTS assay used was also known as Vitamin C Equivalent Antioxidant Capacity (VCEAC). This assay measures the antioxidant capacity through electron donation (Miller & Rice-Evans, 1997). AA has also been shown to possess 1.05 times as much antioxidant capacity as trolox (Huang *et al*. 2005). According to ABTS assay, IC_50_ of AA was 3.464 ppm, and CPHE was 24.10 ppm, which means that it took CPHE approximately 8 times more than AA to possess similar antioxidant activity as AA. Similar to DPPH, this antioxidant capacity of CPHE is thought to be due to the high phenolic content of CPHE (Thaipong *et al*. 2006).

### *In vitro* Assay in HaCaT Cells

#### Cytotoxicity studies of CPHE and AA as cytoprotective agents

Preliminary cytotoxicity studies were conducted on HaCaT cells in order to determine the range of concentration that can be used as cytoprotective agents from the insult against without producing cytoprotective effects on its own. According to previous studies, AA in the form of sodium ascorbate started to inhibit cell proliferation at a concentration of 0.1 mM, and the inhibition progressed as the concentration increased (Savini *et al*. 1999). As it turns out, both CPHE and AA started to exert cytotoxic effects on the HaCaT cells at 100 ppm. Meanwhile, there is limited data on the assessment of cytotoxicity of CPHE on HaCaT cells and other types of cells. CPHE has been found to exert inhibitory effects on various cancer cells, where a wide range of CPHE concentrations ranging from 60 ppm to 800 ppm were shown to exert inhibitory effects (Baharum *et al*. 2014). Another study also showed that CPHE increased cell viability at concentrations between 25 to 100 μg GAE/mL on human umbilical vein endothelial cells (HUVECS) (Felice *et al*. 2020).

It should also be noted that CPHE and AA showed similar cytotoxic effects, even though CPHE showed less antioxidant activity than AA. The antioxidant capacity of CPHE and AA were observed through three different assays, where the mechanisms of action surrounded electron transfer or hydrogen donation. There are many mechanisms of antioxidant properties, and the antioxidant capacity of CPHE and AA are yet to be determined through different mechanisms of actions of antioxidants assays. Other than scavenging of reactive species, other mechanisms of antioxidants include enzymatic activation of intracellular antioxidants, inhibition of enzymes that activate the production of radical species, and many more (White *et al*. 2014).

#### Cytoprotective capacity of CPHE and AA

Though free radicals are not always caused by pollution, it is a good idea to compare the two models together, as H_2_O_2_ represents a more general view on oxidative stress, while cigarette smoke mode represents solely the effects of pollution and observe whether the results are similar or not. Together, these models were used to represent the general pollution model.

Based on the results on Figs. 7 and 8, CPHE possesses a significant cytoprotective potential at 6.25 ppm and 12.5 ppm on H_2_O_2_ and 12.5 ppm, 25 ppm and 50 ppm on CSE. The results also showed that CPHE was able to exert cytoprotective effects at lower or same concentrations as AA, which demonstrated that CPHE possesses comparable antioxidant capacity to that of AA. Different trends were observed in all treatments, which suggests that the cytoprotective activity of both CPHE and AA were not concentration dependent. However, it was observed that unlike AA, CPHE was not able to protect the cells at higher concentrations. This suggests that a different mechanism of anti-pollution effects is used by CPHE.

The mechanisms of antioxidant activity of CPHE is suggested to be due to the phenolic contents. CPHE contains a variety of phenolic compounds, such as protocatechuic acid, kaempferol and flavone derivatives, and other unidentified phenolic compounds (Abdul Karim *et al*. 2014). Phenolic compounds have been previously shown to exert protective effects against oxidative stress and inflammation caused by airborne PM (Rahman *et al*. 2021). This class of compound has also been found to exert antioxidant properties from various mechanisms, including free radical scavenging, enhancing intracellular antioxidant capacity via nuclear factor erythroid 2-related factor 2 (NRF2) activation (Boo 2019). NRF2 itself is a family of transcription factors that regulates the body’s defense system against ROS and RNS, thus the activation is beneficial for protecting the skin (Ma 2013). Another mechanism is by directly inhibiting target enzyme reactions through the inhibition of catalytic activity of protein kinases involved in MAP kinase and NF-κB pathways, as well as metabolic enzymes in prostaglandin synthesis (Boo 2019). On the other hand, vitamin C is well known to exert its antioxidant capacity through radical scavenging, thereby preventing oxidative stress (Carr & Maggini 2017). This suggests that there are other possibilities of antioxidant mechanisms other than radical scavenging for phenolic compounds, explaining why CPHE could still exert cytoprotective effects albeit lower antioxidant capacity measured based on electron transfer or hydrogen donor assays.

During the preliminary cytotoxic studies, AA started showing cytotoxic effects on HaCaT cells with 50 ppm and 100 ppm of concentrations. However, during cytoprotective studies, AA exerted cytoprotective effects against both insult agents on the mentioned concentrations. This result is similar to the previous study (Zhao *et al*. 2008). This difference may be attributed due to the main mechanism of vitamin C antioxidant activity, which is due to radical scavenging activity. Antioxidants that work by scavenging free radicals are neutralised by the free radicals themselves (Lobo *et al*. 2010). This suggests that the free radicals formed were stabilised by AA before it could exert cytotoxic effects on HaCaT cells and rendering the other concentrations not enough to scavenge for free radicals.

Based on the results, CPHE showed cytoprotective effects against pollution and considerable antioxidant capacity. However, the active compound responsible for this effect has yet to be pinpointed. AA was able to exert cytoprotective effects better than the CSE model. This may be attributed to the various skin disease-inducing mechanisms of the CSE model. The mechanisms of PM-induced skin diseases include the hydroxyl and superoxide radical generation, inflammatory mediator production and AhR activation (Moon *et al*. 2020). These mechanisms play a role in producing cytotoxic effects on the cells. The limitation of this study was that the molecular mechanisms of how CPHE and AA exerted their cytoprotective effects were not observed. Therefore, future studies can focus on finding out the molecular mechanism of cytoprotective effects. There are various mechanisms of aging which are caused mainly by free radicals. These mechanisms include: increased matrix metalloproteinase (MMP) which degrades extracellular matrix proteins, impaired transforming growth factor-β (TGF-β) signaling which regulates the synthesis of extracellular matrix proteins, impaired fibroblasts adherence to extracellular matrix (Frangogiannis 2020). Future studies may focus on the expression of these proteins on the skin in order to ascertain the mechanism of cytoprotection. After that, the identification of the phytochemical compound that exerts the highest potency of cytoprotective effects against pollution or other radical-forming compounds on the skin can be researched. This can be done by isolating different fractions of phytochemical compounds found in the extract, which can utilise High-Performance Liquid Chromatography (HPLC), then subjecting the fractions for structural elucidation an identification using detectors such as mass spectrometry and others (Altemimi *et al*. 2017; Sasidharan *et al*. 2011).

### Niosome Formulations and Testing

The formulations used different Span 80 and soy lecithin ratio as the non-ionic surfactant and cholesterol respectively. Several studies suggested different ratios of nonionic surfactant and cholesterol can highly affect the stability and encapsulating efficiency (%) based on the physicochemical properties of the constituents. In this case, a stable formulation with good encapsulation efficiency can increase the penetration capability of the formulation (Fidan-Yardimci *et al*. 2019). Thus, variations were imposed on the formulations described in Table 1 to determine the best ratio of the niosome formulation. The method used was the thin hydration method. Thin hydration method was found to be a simple method compared to the other preparation methods (Thabet *et al*. 2022). In addition, it was suggested that several conditions such as temperature and dissolving time can affect the niosome stability and encapsulation (Zhang 2017). Due to the liquid crystal structure of both soy lecithin and Span 80, temperature higher than the transition temperature (T_c_) of 55°C was needed to ensure the formation of niosome. Thus, a temperature ranging from 55°C–60°C was maintained during the whole thin layer hydration method.

The stability of extract was done for TPC and pH value. Based on the result, the phenolic content decreased around 19% after storage. On the other hand, the result also showed an increase in pH value for the extract after storage. Thus, both results represent the instability and degradation of extract during storage. Phenolic content has an acidic pH value in nature and can be degraded by various reasons such as bacterial degradation and temperature (Friedman & Jürgens 2000). Thus, the degraded phenolic compound will cause a decreased amount of TPC and also lead to increased pH value. All of the niosome formulations were able to retain their pH during storage, proving that the formulation of extract-encapsulated niosome can further stabilise the pH and preserve its content.

The first stability test for the niosome was the encapsulation efficiency using a dialysis bag. According to the principle of dialysis, the amount of encapsulated extract can be determined by measuring the free extract that can pass the membrane. Thus, the lower amount of free extract translates to more encapsulation efficiency. In the result, F1 (4:1) showed the highest encapsulation efficiency at 97.08 ± 3.85% compared to the other formulation, F2 (1:1) and F3 (1:4) with the value of 75.89 ± 3.17% and 67.50 ± 2.39%, respectively. It was suggested that the higher amount of phospholipid leads to higher encapsulation efficiency due to the stabilising mechanism of the phospholipid towards the formulation through hydrogen bond at the niosome bilayer, thus enabling more extract to be encapsulated (Moghassemi & Hadjizadeh 2014; Vu *et al*. 2020). The results were in accordance with these in which F1 (4:1) has the highest amount of encapsulation efficiency with the highest phospholipid ratio. All of the formulations showed a decreased encapsulation efficiency value at day 90 which represent the formulation instability. However, F1 (4:1) still showed the highest encapsulation compared to other formulations with the lowest decrease at 89.88 ± 3.66%. Thus, from both of the results, F1 (4:1) was proven to have highest stability in terms of encapsulation efficiency.

The niosome formulations were further characterised and tested for particle size and surface charge. A surface charge value of more than −30 and +30 mV was considered as a good surface charge for colloidal solution (Joseph & Singhvi 2019). On the other hand, a smaller particle size of the niosome will leadto better penetration overall towards the skin (Yokota & Kyotani 2018). Hence, thegoal was to determine the highest surface charge niosome with the lowest particlesize. From the results, F1 (4:1) has the highest surface charge value of −21.8 ± 0.95 mV and lowest particle size at 375.93 ± 10.75 before storage. However, afterstorage, the result showed a decrease in surface charge value and increase inparticle size due to the instability of the formulations. Despite the instability, F1(4:1) still possessed the highest surface charge value of −19.62 ± 0.72 mV andlowest particle size at 410.16 ± 2.43 nm. Relating the result to the previous study,surface charge of F1 (4:1) still has an inferior value compared to the standardvalue of a good surface charge. The surface charge value of the niosome can be affected by the type of surfactant that was used (Zheng *et al*. 2016). It was suggested that tween type surfactant has a higher surface charge compared to span type surfactants. However, they also reported a less encapsulation result of tween niosome compared to span niosome. In addition, another study showed a smaller particle size in niosome formulated using span compared to tween (Kassem *et al*. 2019). This was due to the lower HLB value which stated to be able to interact with each other at a closer distance, which decreased the particle size of the niosome that will lead to better penetration (Nowroozi *et al*. 2018). Thus, in this study, Span 80 was ultimately chosen due to the lower HLB value compared to tween type surfactants and other span type surfactants (Gupta *et al*. 2011).

Therefore, from all of the characterisation and stability tests of the niosome formulation, F1 (4:1) has the highest stability overall and highest potential to enhance the penetration of the extract.

### Penetration Study of Formulated Niosome

F1 (4:1) showed the highest potential to enhance the penetration of the extract. Porcine ear skin has several characteristics as the best suitable skin model for penetration study in which it has the most similar follicular structure with humans and the thickest amount of epidermis layer (Jacobi *et al*. 2007). In this study, the thickness that was used is 2 mm which has higher thickness compared to the human epidermis layer. Based on the result, all of the formulations have successfully increased the penetration of the extract with F1 (4:1) as the highest cumulative drug release with the value of 83.75 ± 1.37%. In addition, F1 (4:1) was the only formulation that had a significant increase (*p* < 0.5) compared to the control solution at all time points. This relates to the previous test result which suggests that F1 (4:1) has higher penetrating potential due to higher stability and encapsulated extract. Penetration towards the epidermis, especially stratum corneum is limited to passive diffusion (Trommer & Neubert 2006). This leads to low penetration for hydrophilic substances such as extract. However, niosome formulation has higher lipophilicity than extract which enables it to be passively diffused across the layer. Thus, from the mechanism stated, F1 (4:1) has the highest entrapped extract value, which leads to better penetration compared to other formulations.

The amount of penetrated extract was also calculated and further investigated into penetration rate (flux) at specific timepoints. The result showed a similarity towards the cumulative drug release, in which all the formulation has higher flux at all timepoints. However, F1 (4:1) was the only formulation which has a significant difference (*p* < 0.5) compared to other niosomes. On the other hand, the result also showed a decrease in flux at 6 h to 24 h timepoints. Relating to the passive diffusion mechanism, the transfer occurs from high concentrated to the low concentrated environment until an equilibrium is achieved (Cheng & Koskinen 2002). Therefore, at time point 6 h to 24 h the penetration might have reached equilibrium between the donor and receptor compartment of the assay, thus, decreasing the amount of penetrated rate.

Based on these studies, it was shown that F1 (4:1) possessed the highest penetrating capability and flux compared to other formulations, hence it can help the extract to exert antioxidant activity towards the inner skin layer.

## CONCLUSION

It was shown that cocoa pod husk ethanolic extract possesses strong antioxidant capacity, although less potent than AA. The extract showed the best antioxidant capacity through DPPH assay. The extract showed less cytotoxic effects on HaCaT cells than AA, where the extract started exerting cytotoxic effects at a concentration of above 50 ppm, while AA at above 25 ppm (*P* < 0.0001). Both agents were also able to protect HaCaT cells from cigarette smoke extract and hydrogen peroxide insult that was chosen as pollution models in a concentration independent manner (*P* < 0.0001). It was also shown that hydrogen peroxide and cigarette smoke pollution models can be used to represent anti-pollution studies, as both show similar results when studied on AA and CPHE. For the niosome formulations, F1 (4:1) has the highest stability and better characteristic overall as a formulation for the *Theobroma cacao* L. pod husk extract. In addition, this formulation also possessed the highest penetration capability compared to other formulations.

## SUPPLEMENTARY MATERIAL

**Supplementary Table 1:**
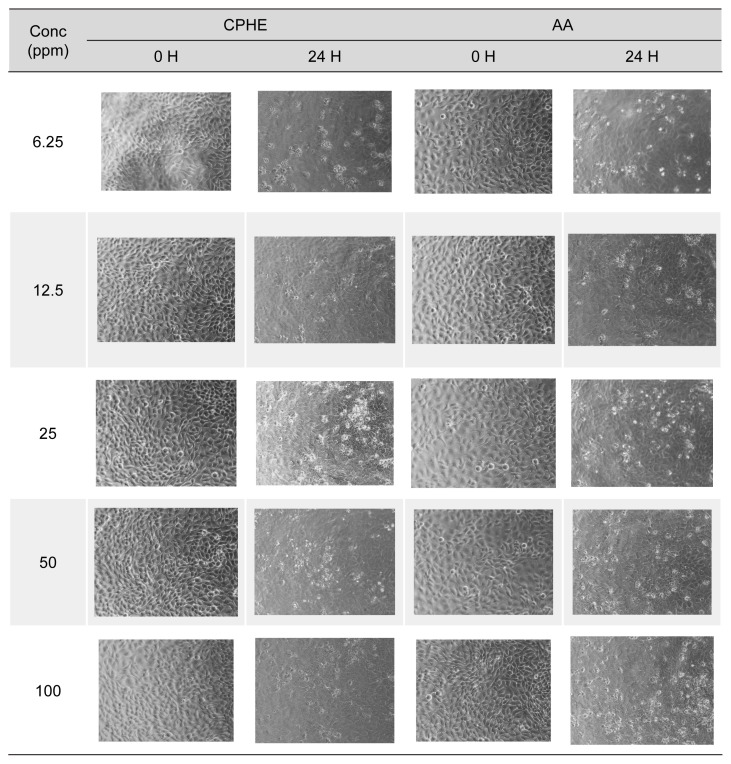
Pictures of HaCaT cells at 0 and 24 h taken under optical microscope after treatment with various concentration of CPHE and AA.

**Supplementary Table 2:**
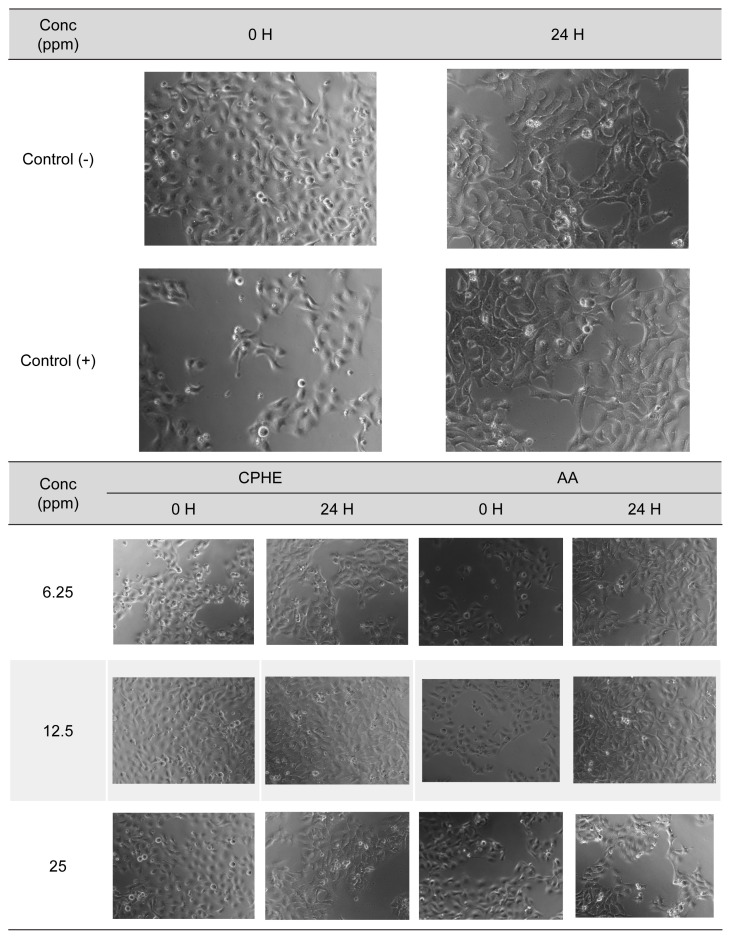

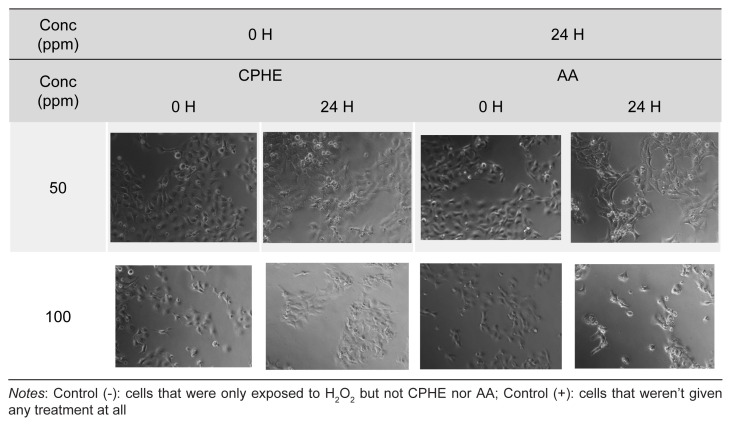
Pictures of HaCAT cells at 0 and 24 h taken under optical microscope after treatment with various concentration of CPHE and AA upon H_2_O_2_ insult.

**Supplementary Table 3:**
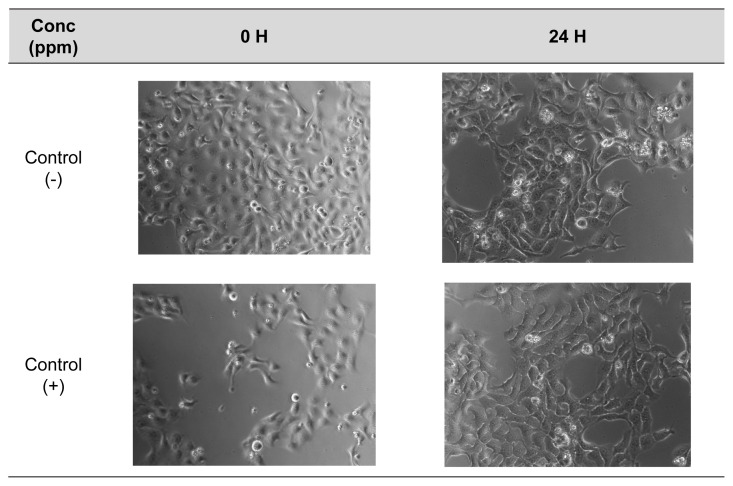

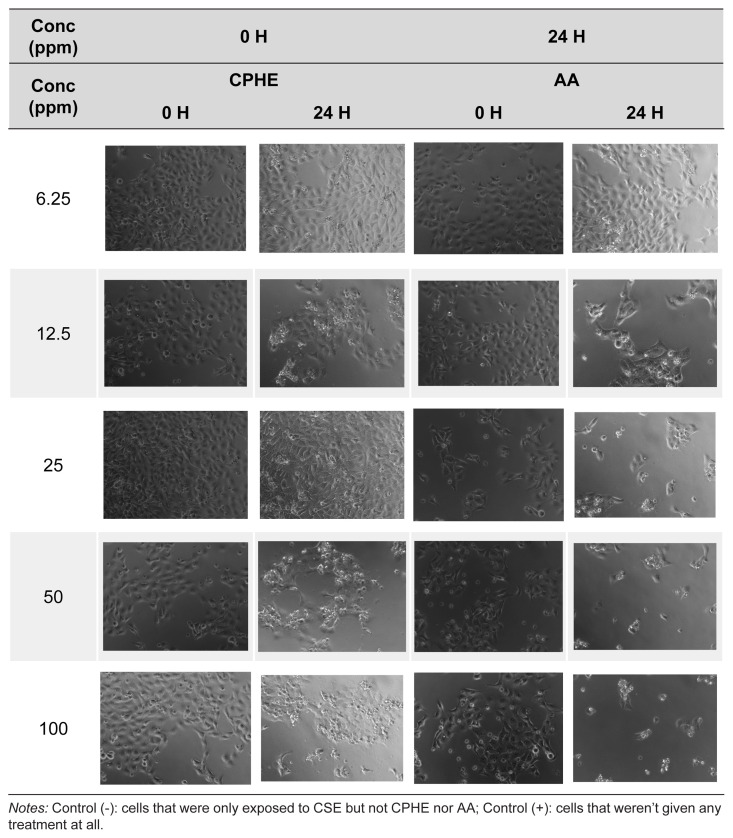
Pictures of HaCAT cells at 0 and 24 h taken under optical microscope after treatment with various concentration of CPHE and AA upon CSE insult.

## Figures and Tables

**Table 1:** Formulation of niosome encapsulated *Theobroma cacao* L.

Ingredients	F1	F2	F3
Extract (mg)	50	50	50
Soy lecithin (mg)	160	100	40
Span 80 (mg)	40	100	160
Hydration solvent	10 mL PBS pH 7.4	10 mL PBS pH 7.4	10 mL PBS pH 7.4

**Table 2:** Antioxidant assay results of CPHE and AA through DPPH, FRAP and ABTS assays.

Assay	Parameter	CPHE	AA
DPPH	IC_50_	19.20 ± 3.59 ppm	13.38 ± 5.96 ppm
	Max inhibition	79.16 ± 1.27%	82.12 ± 0.45%
FRAP	AA equivalent	81.45 mg AA/g extract	–
ABTS	IC_50_	24.10 ± 1.63 ppm	3.46 ± 1.79 ppm
	Max inhibition	93.38 ± 1.12%	98.86%

**Table 3:** TPC, pH, zeta potential, particle size and encapsulation efficiency of all formulations and extract at day 0 and day 90.

Stability	F1 (4:1)		F2 (1:1)		F3 (1:4)	

	0 days	90 days	0 days	90 days	0 days	90 days
pH	7.60 ± 0.02	7.65 ± 0.02	7.32 ± 0.03	7.39 ± 0.02	7.23 ± 0.05	7.38 ± 0
Zeta potential (mV)	−21.8 ± 0.95	−19.62 ± 0.72	−20.1 ± 2.68	−17.92 ± 1.24	−18.26 ± 0.73	−16.56 ± 2.9
Particle size (nm)	375.93 ± 10.75	410.16 ± 2.43	433.76 ± 43.20	530.6 ± 31.80	1008.16 ± 30.17	3924.66 ± 289.06
Encapsulation efficiency (5)	97.08 ± 3.85	89.88 ± 3.66	75.89 ± 3.17	66.70 ± 1.38	67.50 ± 2.39	50.71 ± 10.65
